# Reliable and Efficient Agentic Protein Design with Toolset Harnessing

**DOI:** 10.64898/2026.10.19.759896

**Published:** 2026-10-20

**Authors:** Jasper Butcher, Jie Chen, Andrew Hunt, Cyrus M. Haas, Wenzhao Dai, Shajesh Sharma, Raktim Mitra, Saman Salike, Jeremy Koob, Aiko Muraishi, Zhe Li, Harley Pyles, Michael D. Tyka, Magnus S. Bauer, Rafi I. Brent, Anant Mittal, Neil P. King, David Baker

## Abstract

Computational protein design workflows require the orchestration of multiple tools, data formats, and analysis steps that are often fragmented. Here, we introduce Chaperone, a lightweight and customizable framework that enables seamless integration of RFdiffusion3 (RFD3) [1] with downstream sequence design, structure prediction, and evaluation tools, including ProteinMPNN [2], AlphaFold3 (AF3) [3], and more than 50 other protein design tools. By combining a programmable agent-friendly interface with parallelized execution on high-performance computing systems, Chaperone as a harness enables rapid iteration and scalable exploration of complex protein design workflows. Across an evaluation of nine biomolecular design tasks, we find using Chaperone reduces total token usage relative to baseline agents by 86% and GPU costs by 64%, while increasing the overall computational design pass rate. We demonstrate Chaperone’s utility for large-scale design-space exploration through a two-week end-to-end small-molecule binder design campaign, yielding low nanomolar binders to cortisol zero shot. We further demonstrate its versatility across distinct design challenges, including the design of a symmetric 12-subunit self-assembly nanoparticle and a linker that mediates interactions between C2- and D3-symmetric protein complexes to form crystalline assemblies. Together, these results demonstrate how agent harnesses built around standardized tool interfaces can substantially improve the efficiency and success of complex, long-horizon agentic design workflows in biology. We make Chaperone freely available to the community on GitHub: https://github.com/RosettaCommons/chaperone .

